# Planet of the AAVs: The Spinal Cord Injury Episode

**DOI:** 10.3390/biomedicines9060613

**Published:** 2021-05-28

**Authors:** Katerina Stepankova, Pavla Jendelova, Lucia Machova Urdzikova

**Affiliations:** 1 Institute of Experimental Medicine, Czech Academy of Sciences, Vídeňská 1083, 14200 Prague, Czech Republic; katerina.stepankova@iem.cas.cz; 2 Department of Neuroscience, Second Faculty of Medicine, Charles University, 15006 Prague, Czech Republic

**Keywords:** spinal cord injury, gene therapy, adeno-associated virus, AAV vector

## Abstract

The spinal cord injury (SCI) is a medical and life-disrupting condition with devastating consequences for the physical, social, and professional welfare of patients, and there is no adequate treatment for it. At the same time, gene therapy has been studied as a promising approach for the treatment of neurological and neurodegenerative disorders by delivering remedial genes to the central nervous system (CNS), of which the spinal cord is a part. For gene therapy, multiple vectors have been introduced, including integrating lentiviral vectors and non-integrating adeno-associated virus (AAV) vectors. AAV vectors are a promising system for transgene delivery into the CNS due to their safety profile as well as long-term gene expression. Gene therapy mediated by AAV vectors shows potential for treating SCI by delivering certain genetic information to specific cell types. This review has focused on a potential treatment of SCI by gene therapy using AAV vectors.

## 1. Introduction

A spinal cord injury (SCI) is damage to the spinal cord. Injury consequences cause temporary or permanent changes in spinal cord function. Symptoms may include the loss of motor, sensory, and autonomic nervous system functions immediately after the injury, and later problems such as muscular atrophy, chronic pain, and urinary infection, in parts of the body which are present below the lesion level [1,2] of the spinal cord.

Traumatic spinal cord injury is one of the most devastating kinds of injury. It may lead to different degrees of paralysis, sensory loss, and dysfunction of the bladder. It not only affects the patient’s health, but affects daily life, identity, and society role; it also generates a huge economic burden on the family and society [1,2]. According to data of the World Health Organization (WHO), between 250,000 and 500,000 new cases are reported every year. The majority of spinal cord injuries are caused by trauma such as road traffic accidents, falls, or violence. People with a spinal cord injury are two to five times more likely to die prematurely. The worst survival rate has been shown in low- and middle-income countries. Spinal cord injury is also associated with lower rates of economic participation and school enrollment, along with societal costs.

Despite the severe consequences for spinal injured patients and their families, and healthcare costs being among the highest of any medical condition, there is still no cure or adequate treatment, even though there are countless experimentally successful approaches to improving neurological functions in animal models.

During the past several years, adeno-associated virus (AAV)-mediated gene therapies have achieved clinical interest for treating a large range of neurodegenerative and neuromuscular diseases, such as: amyotrophic lateral sclerosis [3], Alzheimer’s disease [4,5,6,7], Parkinson’s disease [8,9,10], Huntington’s disease [11], spinal muscular atrophy [12], Pompe’s disease [13,14,15,16,17], aromatic L-amino acid decarboxylase deficiency [18], Friedrich’s ataxia [19], etc. In experimental spinal cord injury settings, gene therapies were used to break down scar tissue and enable new growth and reconnections between the spinal cord and muscles, or between the spinal cord and brain, that control motor movements and sensation [20,21,22,23,24]. AAV vectors have demonstrated the potential solution for the above-mentioned diseases, by delivering a functional copy of a gene into the nucleus of the somatic cells in the affected tissues [25].

This review provides the bridge to our understanding of the capsid virology of different AAVs, their potential in spinal cord injury treatment, and preclinical studies demonstrating their use in rodent models of SCI. However, the AAV-mediated gene therapy of SCI is still in its infancy; thus, there is no successful clinical translation yet and research in this field requires further work and optimalization.

## 2. Spinal Cord Injury

A spinal cord injury is damage to any part of the spinal cord that often causes permanent changes in sensation, strength, and other body functions below the site of the injury. In general, there is a relationship between the level of completion of the injury and its functional status, along with the location of the lesion within the spinal cord. Therefore, the patient’s ability to control body movement and to feel, depends on the severity (known as completeness) and the place of the injury. It usually applies that a higher located injury causes more severe consequences, and the more severe the injury, the more likely the result will include permanent impairment.

### 2.1. Primary Injury

SCI is initiated by a mechanical trauma where compression forces and traction are involved. Direct compression of the spinal column leads to the damage of neural elements by displaced and fractured bone fragments, disc material, and ligament injures [26]. Axons are disrupted, blood vessels damaged, and neural-cell membranes broken. Within a few minutes, the spinal cord swells at the injury level, and occupies the entire width of the spinal canal. If the spinal cord swelling outpaces venous blood pressure, it will result in secondary ischemia [26,27]. The autoregulation of the blood flow then fails. The spinal neurogenic shock manifests as systemic hypotension, leading to intensified ischemia. Further disruption of neuronal membranes occurs, and toxic chemicals are released. An electrolyte imbalance triggers a secondary injury cascade. It considerably adds to the initial mechanical trauma by killing or impairing the surrounding cells [28].

### 2.2. Secondary Injury

Secondary events are initiated by the primary injury [29,30]. After the injury, the hypoperfusion that develops in the gray matter expands to the white matter that encloses the gray matter. The action potential propagation along the axons is slowed or fully blocked after the hypoperfusion, contributing to spinal shock [31].

The damaged cells and blood vessels release toxic chemicals. The nervous system is vulnerable to them. These released toxins attack the intact surrounding cells. Glutamate plays a crucial role in a highly troublesome process called excitotoxicity [32]. In the healthy spinal cord, axonal terminals are specialized to secrete glutamate. The excretion of the neurotransmitter is highly regulated in the body. Glutamate binds to receptors on target neurons and stimulates these cells to fire impulses. On the other hand, glutamate floods out of the injured spinal neurons and astrocytes, and overstimulates the surrounding neurons. The overexcited cells release calcium ions that trigger a series of destructive events and biochemical cascades, including the production of free radicals, ionic imbalance, and cell apoptosis [30,33]. Axonal growth is inhibited by inhibitory molecules associated with myelin, and molecules associated with the ECM. Myelin is produced by oligodendrocytes. When myelin-producing cells die, the uninjured axons become demyelinated, and they are therefore incapable of conducting impulses after SCI. The demyelination and myelin disintegration release myelin-associated inhibitors, namely, myelin-associated glycoprotein (MAG), oligodendrocyte myelin glycoprotein (OMgp), and neurite outgrowth inhibitor, A (Nogo-A) [34]. These molecules are localized on the membranes of oligodendrocytes. MAG, OMgp, and Nogo-A have almost nothing in common except binding to the Nogo receptor (NgR1) and Paired immunoglobulin-like receptor (PirB). Myelin-associated molecules-NgR1-mediated inhibition activates cytoskeletal changes, leading to axonal growth inhibition. The signaling cascade involved in NgR1 mediated inhibition will be discussed in more detail in Section 4.3. In contrast, the inhibition involving PirK triggers an intracellular signaling pathway, but the mechanism of cytoskeletal changes it causes is not fully understood [35].

**Figure 1:**
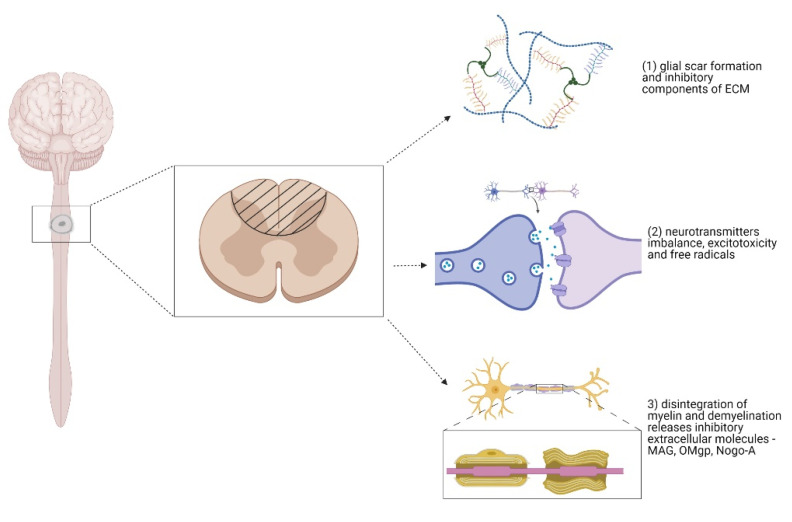
Spinal cord injury pathology. The regeneration following traumatic spinal cord injury (SCI) fails especially due to: (**1**) glial scar formation and changed extracellular matrix (ECM) environment caused by secretion of tenascin, chondroitin sulfate proteoglycans (CSPGs) such as brevican, phosphacan, neurocan, versican, and NG2 proteoglycans. All of these molecules lead to the activation of Rho-ROCK signaling pathway inhibiting regeneration; (**2**) Neurotransmitter imbalance, excitotoxicity, and free radicals release caused by overstimulation of cells; (**3**) inhibitory molecules associated with the ECM and those associated with myelin such as MAG, OMgp, Nogo-A released after myelin disintegration (Created with BioRender.com).

After the SCI, glial cells, fibroblasts, and macrophages are activated to restrict the spread of inflammation and protect spared neural tissue [36,37]. The glial cells that form the glial scar play a key role in spinal cord regeneration. The glial scar is formed by reactive astrocytes, macrophages, microglia, fibroblasts, and oligodendrocyte precursor cells, also called NG2. NG2 glia cells are an important part of the scar; they migrate to the site of injury where they are a source of NG2 chondroitin sulfate proteoglycan (CSPG) for the extracellular matrix [38]. The ECM of the glial scar mostly contains CSPGs, with heparan sulphate proteoglycans (HSPGs), tenascins, and molecules bound to them [39]. The proteoglycan gradient increases towards the scar center. However, the glial scar plays a dual role in SCI, both protective and inhibitory [40]. The glial scar inhibits an axonal regrowth by mechanical blockage through inhibitory molecules and ECM components, due to the astrocytic upregulation of CSPGs after SCI. The other cells (NG2-OPCs, macrophages, oligodendrocytes) also contribute to CSPGs enrichment. The beneficial role of the glial scar restricts the spread of inflammation by limiting the detrimental effects of fibrotic tissue and macrophages, thus, protecting spared neural tissue. Moreover, since the glial scar is mostly formed by newly proliferated astrocytes with immature astroglia properties, this would support axonal regrowth across the lesion. Figure 1 shows the main reasons for the axonal regeneration failure [41,42,43,44].

## 3. AAV Vectors as Tools to Repair Injured Spinal Cord

### 3.1. AAV Biology

AAVs are members of the family Parvoviridae. They are non-pathogenic, non-enveloped, helper-dependent animal viruses with icosahedral capsid architecture composed of 4.7 kb single-stranded DNA genome. The genome contains three open reading frames (ORFs). The ORFs are surrounded by inverted terminal repeats (ITRs) on the 5′ and 3′ ends, working as replication origin and packing signal. The first ORF, rep, encodes four non-structural proteins playing an important role in viral replication, transcriptional regulation, genome integration, and virion assembly. The second ORF, cap, encodes three structural proteins (VP 1–3) to form a viral capsid. The third ORF is presented as an alternate reading frame overlapping the *cap* gene. This region of the DNA allows the translation of an assembly activating protein, one of the viral capsid proteins. This protein localizes AAV capsid proteins to the nucleolus and takes part in the capsid assembly process [45,46,47].

Over the past few years, 12 natural serotypes have been found and isolated from humans and other primates [48]. These serotypes differ in their tropism, or the types of cells they infect. Serotypes make an AAV viral vector, specific for transducing a certain cell type. Different serotypes are defined by distinct capsid protein motifs, determined by different neutralizing antibodies [49]. Each serotype shares the same set of antigens on their cell surface. These antigens react to a specific set of neutralizing antibodies. Neutralizing antibodies which are reactive to the capsid proteins of one serotype cannot react to the viral capsid of another serotype [50].

Furthermore, AAV serotypes present the characteristic properties involved in the capsid and receptor interaction. These differences in the cell receptors that they recognize determine the cell type and tissue type tropism of AAV serotypes [51,52]. Several studies have compared the transduction of AAV serotypes in different tissue. However, it is quite difficult to interpret the data due to variations in vector titers and doses, as well as transgenes, and promoters, and last but not least, due to a general hierarchy of transduction efficiency in major tissues. It has been established that AAV1, AAV2, AAV4, AAV5, AAV8, AAV9 are optimal serotypes for CNS; AAV1, AAV8, AAV9 for heart; AAV2 for kidneys; AAV7, AAV8, AAV9 for liver; AAV4, AAV5, AAV6, AAV9 for lungs; AAV8 for pancreas; AAV2, AAV5, AAV8 for photoreceptor cells; and AAV1, AAV6, AAV7, AAV8, AAV9 for cytoskeletal muscles [53].

The capsid allows the transduction of cells, from the first contact with a cell surface receptor to the entering of the nucleus. The AAV attaches to its specific receptor in the membrane. When the virus is bound to the receptor, it enters the cell through clathrin-coated tips [54]. It escapes from the endosomal pathway, and transits to the perinuclear area. Upon arrival, it gets into a cell nucleus, the virus is uncoated, and its single-stranded genome is released, then the second strand is synthesized and finally, transcribed [46,55]. After cell infection, the virus may follow the lytic or the lysogenic cycle pathway. The AAV needs a co-infection with a helper virus to replicate and complete its life cycle. If there is a helper virus (e.g., adenovirus), the AAVs engage in the lytic cycle which leads to the rapid replication and release of new viral particles into the environment. In the lytic cycle, after the attachment to the cell and following penetration, the biosynthesis of viral DNA and viral proteins starts and, when new phages are assembled, the lysis of the host cell occurs. The host cell is destroyed, and new viral particles are released. If there is not a helper virus, the viral genome gets into the host cell, where most copies are eliminated after a short time, but some of these copies persist long-term in a latent form [56]. In contrast, in the lysogenic cycle, the host cell is not destroyed but the virus integrates into the chromosome or maintains its genome as extrachromosomal episomes and becomes part of the host cell. Due to its integration into DNA, it is replicated during the cell cycle with the host cell and passes into the new daughter cells. Latent copies of the AAV genome persist in an episomal and/or circular form, and they can be reactivated with a helper virus leading to the progeny viral progeny production [57].

In recombinant versions of AAV (rAAVs), the gene of interest is put inside between the ITRs [58], and rep and cap are supplied *in trans.* rAAVs requires helper viral genes during vector production. The resulting vector can transduce both nondividing and dividing cells with a stable transgene expression, in the absence of a helper virus in post-mitotic tissue for years [25].

### 3.2. AAV as a Vector for In Vivo Gene Therapy

#### 3.2.1. RAAV Technique

Wild-type AAV (wtAAV) preferentially integrates into the specific genomic site on human chromosome 19, due to Rep sequence and Rep proteins. The rAAVs are constructed with Rep protein supplied *in trans*. As a result of this the superinfection, in the event of the presence of helper viruses, cannot appear as rAAVs are not able to integrate into the wtAAV’s preferential integration and trigger long-term transgene expression in post-mitotic tissues, most likely because they persist as episomes within the host cell [59]. rAAV vectors are composed of the *cis* element ITRs. These sequences are the ones that rAAVs have in common with the viral original genome. ITRs are needed to instruct genome replication as well as packaging during vector production. The rest of the wtAAV protein-coding sequences are replaced with the therapeutic gene expression cassettes. The rAAV´s genome is encapsidated within a serotype-specific capsid. When the viral coding sequence is completely replaced, it boosts the packaging capacity of rAAVs, as well as contributing to their low cytotoxicity and immunogenicity after in vivo delivery [60,61].

At present, rAAV vectors are designed with a broad spectrum of transgene cassettes and promoter elements for different gene transfer applications. The most crucial factors determining the AAV vector efficiency are serotypes, as mentioned in the previous text, and promoters. The promoter is a sequence of DNA regulating gene expression. For the AAV construct, the right promotor must be chosen to achieve a sufficient level of gene expression which leads to regeneration. A promoter drives gene expression when the gene is delivered to the appropriate cell. There are two types of promoters: tissue-specific promotors activating only the specific cell types, and ubiquitous promoters promoting gene expression in more than one cell type [60]. Promoters consist of the core/proximal promoter located upstream of the regulated gene and convey the transcription factor binding site [62]. At this point, promoters may be constitutively active or derived from viruses [63]. Constitutively strong promoters include the spleen focus forming virus (SFFV), the mouse phosphoglycerate kinase (mPGK), the human polypeptide chain elongation factor 1α (EF1α), the chicken beta-actin (CBA) promoters or the ubiquitin C (UbiC), the human cytomegalovirus (hCMV). These promoters provide rapid, robust, and long-term transgene expression in a variety of cell types [64]. As a neuron-specific promotor, a small fragment of the human synapsin 1 (hSYN) gene promotor can be used, with stable and long-term transgene expression from its vector [65]. Promoters can also be constructs combining one or several of these cis-regulatory elements. This combination can provide a very potent expression cassette, but the biggest disadvantage here is their limited packaging capacity. Therefore, shorter promoters are developed, by deleting non-essential parts and preserving a high level of tissue specificity and gene expression [66]. An example of the ubiquitous hybrid promoter is a short variant of CAG (sCAG) composed of the CMV immediate-early enhancer, the chicken β-actin (CBA) promoter, and the CBA intron/exon 1 [67]. A short variant was made when the full-length version failed to reach the expression levels in the brain and motoneurons [68]. The study of Nieuwenhuis and his colleagues in 2020 compared four promotors, sCAG, hCMV, mPGK, and hSYN promoter, in their capability to initiate the transgene expression of cortical neurons. The results have shown that the mPGK and hSYN promotors led to the strongest transgene expression, in comparison with hCMV and sCAG. In addition, the hSYN promotor was the most specific in neurons, leaving the glial cells intact [69].

#### 3.2.2. Clinical Use of AAV-Based Gene Therapy

The AAV based gene therapy has already been tested for neurodegenerative disorder treatment in several clinical applications. Table 1 summarizes active and already completed clinical trials in February 2021.

AAVs are not the only viruses used for gene therapy, but it has been shown that they have a whole range of advantages from superior biosafety (which refers to the protection of public health and environment from accidental exposure to the viruses) rating and biosecurity (which refers to the prevention of misuse or intentional release of viruses) of rAAVs through stable expression to low immunogenicity, and a very mild response in vivo [25,47,70]. Thus, AAVs are relatively simple from an immunogenicity point of view. rAAVs do not contain any viral genes as they comprise a DNA genome and a protein capsid, hence, there will be no active viral gene expression to trigger and amplify the immune response [71].

However, there is always an alternative perspective, and even AAVs have their disadvantages. AAVs have a cloning capacity limit, which means they cannot be used for larger gene delivery [72] and, in spite of their low immunogenicity, they can generate neutralizing antibodies which may weaken their positive effect [73].

The existence of pre-existing immunity starts when the organism is exposed to wtAAV. It can generate both cell-mediated and humoral immune response to the virus [74]. wtAAVs are highly prevalent in the human population [48], even though exposition to these viruses have not clearly been associated with any disease or clinical pathology [75]. After primary infection, the wtAAV genome can persist in host cells for several years as an episomal, for example, and it may be reactivated by a helper virus [76]. The immune system reacts to the virus by producing neutralizing antibodies for defending cells from pathogens, and non-neutralizing antibodies binding specifically to virus particles [77]. The presence of these antibodies can lead to a cross-reaction with the AAV vectors after systematic delivery [78]. Therefore, pre-existing humoral immunity has been considered to be the biggest obstacle for the successful gene transfer through the systemic administration of rAAV [78]. However, the immune response to rAAVs is still not fully understood.

It has been proved that AAV-specific memory T cells produce interleukin 2 (IL-2), interferon gamma (IFNγ), and tumor necrosis factor alpha (TNF-α). These cells also present a cytotoxic phenotype. It is characterized by the expression of CD107a degranulation markers and granzyme B [79].

In the simplest term, when the human body has been exposed to wtAAV before AAV vector administration, the vector components can trigger an innate immune recognition. There are some observed cases of pyrexia, besides complement activation. Following vector administration, antibodies against AAV capsid are produced and remain for several years after gene transfer. In some cases, the immune response even correlates directly with the loss of transgene expression. The immune response to vector or transgene could be a potential immune-related barrier in gene therapy [80]. Immune responses documented in human patients and in animal models have suggested how to solve this obstacle. The efforts to further reduce vector immunogenicity might be accomplished by ITRs modification. ITRs contain CpG island innating immune response via TLR9, as they are presented by MHC I. It is suggested that the immune reaction could be reduced by CpG depletion [81,82].

An important step for transferring preclinical investigations to clinical studies is research on larger animals such as dogs, cats, pigs, and non-human primates (NHPs). One of the reasons why studies on larger animals, especially NHPs, are essential is the different immune reaction when compared to mice and rats. The main difference is that humans and NHPs are natural hosts for AAVs compared to rodents, and it is not possible to fully reproduce the reaction of memory CD8+ T cells in murine models, as was explained by Herzog et al. [83].

It has also been shown that the transduction of different parts of the CNS, when AAVs are injected intrathecally, differs across animal species. In rodents, intrathecal AAV delivery produces only minimal brain transduction [84,85,86], while using pigs as an experimental model seems to be more promising at the level of brain transduction and the intrathecal injection [87]. In NHPs, the injection of AAV9 into the cisterna magna resulted in widespread brain transduction, like intravascular delivery [87,88]. We can speculate that the variability in size and anatomy in different species plays a role. For example, when we compare humans, primates, and pigs, porcine epidural space contains fatty deposits that may restrict the CSF flow. The biodistribution of AAVs is also an important parameter. After an IT injection of AAV9 in pigs, the transduction of peripheral organs was barely detectable, whereas in NHPs, the vector was detected in the liver and spleen at levels that equaled or exceeded levels seen in the CNS [87]. Further investigation is needed to explain if the differences in biodistribution are caused by physiological/anatomical differences, or differential receptor biology and binding kinetics of AAVs.

**Figure 2:**
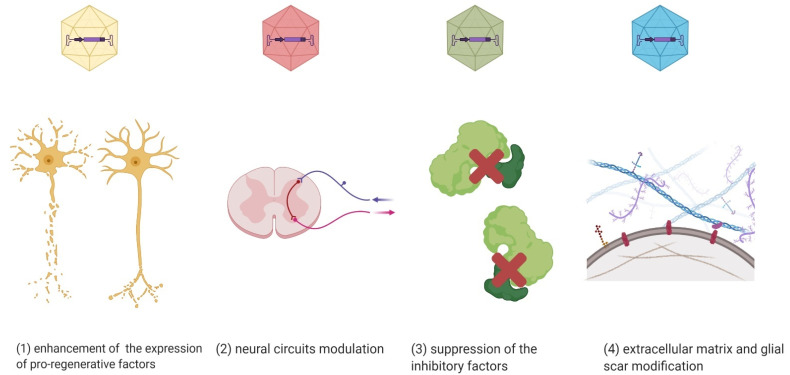
Gene therapy applications shown in preclinical SCI models. AAV vectors can express (**1**) pro-regenerative factors, (**2**) circuit-modifying factors, (**3**) repressors for inhibitory factors, and (**4**) matrix/glial scar-modifying factors (Created with BioRender.com).

Currently, there are no clinical trials using AAV vectors in SCI treatment. However, there are experimental and preclinical studies using in vivo AAV-mediated gene therapy in the injured spinal cord to (1) enhance the expression of pro-regenerative factors, (2) modulate neuronal circuits, (3) suppress the inhibitory factors and apoptosis, and (4) modulate an extracellular matrix or/and glial scar, as well as the cytoskeleton (Figure 2) [89].

### 3.3. AAV Administration into the Spinal Cord

**Figure 3:**
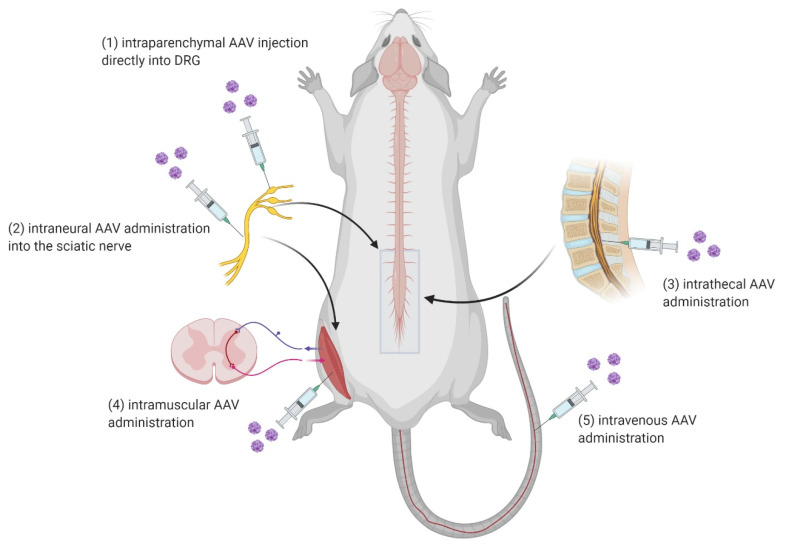
Possible routes of AAV vector administration into the spinal cord: (**1**) intraparenchymal, (**2**) intraneural, (**3**) intrathecal, (**4**) intramuscular, and (**5**) intravenous AAV delivery (Created with BioRender.com).

AAVs can be delivered remotely or directly. In general, there are several routes of administration. Remote delivery includes several non-invasive or minimally invasive routes, relying on the AAV serotype specificity and ability to get from the periphery to the spinal cord, such as intramuscular (IM), intraneural (IN), and intravenous (IV). Intrathecal (IT) routes of administration can also be considered minimally invasive when the viral vector is supposed to be delivered to brain cells. After the viral injection directly into the cerebrospinal fluid (CSF), rAAV can cross the blood–brain barrier and the expression can go straight to the brain cells without the need for direct administration in the brain [90]. Direct delivery, on the flip side, involves an invasive surgical intervention that targets a specific area of the spinal cord tissue. This intervention is known as an intraparenchymal way of a viral vector delivery (Figure 3) [91].

IM and IN routes of administration use the axonal transport machinery to bring vectors from peripheral injection sites to the corresponding neuronal cells in the spinal cord. IV administration has shown the capability of AAV vectors to cross the blood–brain-barrier (BBB) and become increasingly promising in therapeutic gene delivery to the spinal cord [92]. The IM route includes viral interaction with the muscle cell surface; the cell enters and then internalizes at the nerve terminals. This process is highly regulated by endocytic pathways. The virus uses the Rab GTPase-mediated endosomal sorting system. Endosomes are further transported retrogradely along microtubules. Gene expression starts when the virus enters the neuronal nucleus successfully [93].

The IT route of administration means direct delivery of the viral vector substance into the subarachnoid space to reach CSF. It allows the diffusion and penetration into the spinal cord parenchyma. The main difference between the direct administration and the remote delivery of viral vector has been shown especially in the ability to precisely target specific regions of the spinal cord and in the lower volume of the AAV substance required in direct administration [91,92].

## 4. Using AAVs to Help Recovery after Spinal Cord Injury: Preclinical Results

### 4.1. The Expression of Pro-Regenerative Factors

**Figure 4:**
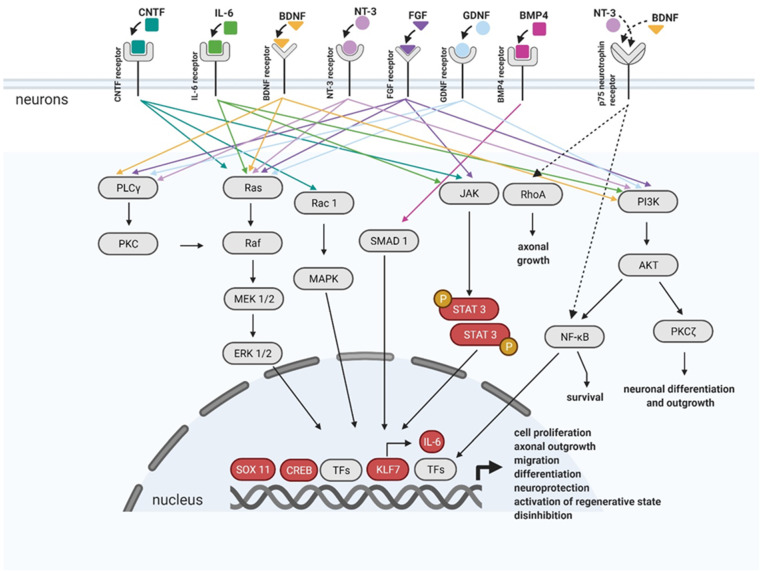
Schematic representation of connections between pro-regenerative factors and transcription factors (TFs) targeted in AAV-mediated gene therapy. Pro-regenerative factors and TFs mentioned in the text above the figure are marked in colors (Created with BioRender.com).

Matured CNS has poor neuronal intrinsic growth ability. This is the reason why axons cannot regenerate and why there is no functional recovery. Many earlier studies have already shown that the neurites in the early postnatal spinal cord can regenerate more easily after SCI in comparison to matured CNS [94]. One of the approaches of how to cure the SCI is thought to be the recapitulation of some of the developmental processes that occur before processes such as synapse formation, and neuronal activation [95] (Figure 4).

One of the easiest ways to recreate a developmental environment in the matured CNS is by inducing the expression of the neurotrophic factors via AAV viral vectors containing the neurotrophic factors genes. One of them is a Ciliary neurotrophic factor (CNTF), a neuropoietic member of the interleukin 6 (IL-6) cytokine family [96] that can promote survival and enhances long-distance regeneration of injured neurites in parts of the adult CNS. CNTF signals work through the Janus kinases (Jak)/signal transducer and activator of transcription proteins (STAT) signaling pathway. CNTF has been used to increase the regeneration capacity of rodent adult retinal ganglion cell axons, and it promoted extensive and successful regrowth of axons [97]. The delivery of the AAV encoding and expressing a secretory form of CNTF into the cortical regions of the brain projecting onto the outputs of the corticospinal tract (CST) allows the expression of CNTF in neurons, including corticospinal neurons (CSN), after Th level hemisection and contusion SCI studies [98,99,100].

The family of neurotrophins consists of structurally related proteins. These proteins bind to the tropomyosin receptor kinase (Trk) receptor, but all members can bind with lower affinity to the p75 (NTR) receptor [101]. It has been shown that changing the levels of neurotrophins in the spinal cord microenvironment after SCI has led to normal function recovery, as an improvement in chronic pain response to peripheral stimuli and locomotor functions [102]. Brain-derived neurotrophic factor (BDNF) is crucial for synaptogenesis, neurite outgrowth, synaptic plasticity, and synaptic transmission during development [103]. After transection at the Th9-10 and injection of AAV-BDNF at the level Th11-12, rats significantly showed improvement in plantar stepping, which started shortly after complete transection [104]. Neurotrophin-3 (NT-3) has also been extensively used in experimental SCI treatment. NT3 plays a key role as a survival and trophic factor during development and after injury [105]. An intramuscular injection of AAV-NT3 to the musculus triceps brachii increased the number of CST fibers in C4–5 lesioned rats beyond the lesion site. It also led to a milder functional loss after SCI [106]. Furthermore, an intramuscular injection into each rat’s bilateral tibialis anterior and soleus muscles after contusive injury at Th9 level, and regular exercise, alleviated muscle spasms by modulating the excitability of spinal neurons and motor neurons [107].

Other growth factors, such as fibroblast growth factor (FGF) belonging to a large family of heparin-binding proteins interacting with membrane-associated proteoglycans, are present in the CNS and PNS during development and throughout the lifetime. FGFs stimulate neuronal cell fate determination, migration, and differentiation. FGFs bind and activate fibroblast growth factor receptors (FGFRs). Activated FGFRs trigger several signaling pathways leading to specific cellular responses. Well studied pathways are the RAS/MAP kinase pathway, PI3 kinase/AKT pathway, and PLCγ pathway [108], schematically shown in Figure 4. AAV mediated FGF1 overexpression in the lesion site after T9 contusive injury resulted in improved motor functional recovery of SCI rats [109]. In another study, FGF1 facilitates neuroprotection, axon regeneration, and remyelination after AAV-FGF1 administration into the lesion area, immediately after SCI in a rat model [110]. FGF2 acts like a regulative response to CNS injury, including the transformation of reactive astrocytes and neurogenesis. Furthermore, FGF2 has also been examined in SCI regeneration. Several studies have shown an enhanced CST growth, when AAV-FGF2 had been delivered close to the injury site after SCI [111,112,113].

It has also been shown that a glial cell-derived neurotrophic factor (GDNF) overexpression, mediated via AAV-viral vector, significantly debilitated the death of spinal cord ventral horn motor neurons [114]. GDNF is a factor that protects neurons from various stresses, and it may potently promote the survival of many types of neurons [115]. GDNF can activate the MAPK/Erk pathway as well as P13K/AKT pathways, by binding to the receptor tyrosine kinases (RTKs) [116,117]. Moreover, membrane microdomains known as lipid rafts are essential for the GDNF signaling pathway [118]. Ret tyrosine kinase localized in the membrane lipid rafts interacts with members of the Src-family kinases through its receptor, which is necessary for neurite outgrowth and survival [118,119].

There is growing evidence that a combination of growth factors has a greater effect on axonal regeneration. Multiple genes can be combined into one viral construct [120]. This fact makes the administration even easier, prospectively combined with exercise (previously mentioned) or electrical stimulation [121].

Injury response is controlled in axons by the regeneration associated gene (RAG) program which is, however, insufficient and can therefore cause the failure of neurons to regenerate. The viral vector-mediated gene transfer became a powerful strategy on how to manipulate the expression of transcriptional factors (TFs) needed for RAG response in injured neurons [95]. The expression of RAG genes is triggered by an intrinsic molecular mechanism involved in the physiological regenerative response [122]. The RAG program includes hundreds of genes and regeneration-associated transcription factors (TFs). To facilitate regenerative capacity, the AAV-mediated gene therapy associated with the RAG program targets either regeneration-associated TFs (such as CREB, KLF7, SOX11, etc.) or terminal RAGs (such as BDNF, GDNF, IL-6, etc.) upregulation to enhance the RAG program response [123,124].

The overexpression of constitutively active cAMP Response Element-Binding Protein (CREB) in sensory neurons located in DRG increased the regeneration after dorsal column crush [125]. The overexpression of Signal transducer and activator of transcription 3 (STAT3) enhanced the speed of outgrowing axons and the promotion of collateral sprouting after SCI in mice [126]. STAT3 AAV gene therapy also enhanced the sprouting and remodeling of injured corticospinal neurons which led to functional recovery [127]. The delivery of transcriptional active Krüppel-like factors 7 (KLF7) promoted axonal regeneration in CST [128,129]. KLF7 has a multifunctional effect; it regulates the expression of p300, activates the expression of interleukin 6 (IL-6), and activates the Suppressor of Mothers Against Decapentaplegic homologue 1 (SMAD1) via Bone morphogenetic protein 4 (BMP4). Adenoviral-mediated overexpression of BMP4 in DRG neurons enhanced the sprouting of dorsal column axons after SCI [130]. In addition, the trans neuronal application of hyper IL-6 (AAV-hIL-6) promotes functional recovery. A cortical AAV2-hIL6 injection triggered STAT3 activation and improved neurite regeneration in CST. Virally expressed hIL-6 was released from the soma to stimulate adjoining motoneurons due to the paracrine effect, but the signal was also measured over long distances in axons of retinal ganglion cells and cortical neurons. The recovery was assessed by a behavioral test and immunohistochemistry [131]. These findings suggest a new approach for combining strategies to further improve functional recovery, for example, by modifying the micro-environment around the lesion site by alleviating the postinjury tissue destruction or secondary damage and glial scar formation [132].

Another pro-regenerative gene therapy targets Sry-type HMG box 11 (Sox11) transcriptional factor. During periods of axonal growth in the embryonic CNS and PNS, Sox11 is widely expressed and then is developmentally downregulated. Sox11 is functionally involved not only in the developing nervous system, but it also plays a role in tumorigenesis and adult neurogenesis. It has been discovered that a viral-induced expression of Sox11 reduces the axonal dieback of DRG axons and promotes CST sprouting. The mechanism of how Sox11 works on the molecular level is relatively unknown, but it may promote axon growth in CST neurons by activating pro-regenerative genes such as small proline-rich protein 1a (Sprr1a), BDNF, TNF receptor associated factor (TRAF), and a family member-associated nuclear factor-κB activator (TANK) [133].

### 4.2. Modulation of Neural Circuits

Another approach of how to improve the lives of patients after SCI is to reconfigure neuronal circuits. This strategy is based on anatomical changes which lead to environmental reconstruction and specify the functional circuits that give rise to a certain behavior. It may be beneficial for SCI patients, for assisting basic functions such as respiratory problems and bladder control. Neural circuit modulation aims to circumvent the lesion core and reform functional circuits, hence activating the already existing neural tissue in a dormant state [134]. Chloride potassium symporter 5 (KCC2) is an important modulator of neural circuits. KCC2 makes a significant contribution to inhibitory neurotransmission (Hyperpolarizing GABAergic transmission is KCC2-dependent) and consecutively balances the ratio between inhibitory and excitatory synapses. AAV is an elegant way to attain the expression of KCC2 and modify the spinal neural circuits. The AAV mediated KCC2 overexpression resulted in functional recovery assessed by the behavioral test. Motor functions were evaluated with the Basso Mouse Scale (BMS), a locomotor open field rating scale, and for the skilled walking assessment a ladder rung walking test was used. The functional recovery became significant from 7 weeks after treatment [135]. Moreover, it is known that KCC2 are downregulated after the SCI. It causes less-negative equilibrium potential for Cl¯. Thus, neurons have increased excitability related to the elevated probability of action potential generation in synaptic excitatory response [136]. The downregulation of KCC2 also leads to hyperalgesia [137]. In the spinal cord injured mice model, KCC2 overexpression showed neuropathic pain reduction [138].

A highly significant way to manipulate only certain neurons is to target the cell bodies of projection neurons. The AVV method is not pathway-specific, but its specificity may be enhanced by coupling with neuromodulators (e.g., a DREADD or opsin) [139]. The DREADD abbreviation stands for Designer Receptors Exclusively Activated by Designer Drugs, which are artificially engineered protein receptors. They can be used as a chemogenetic tools in biomedical research. The chemogenetic tools can be used by neuroscientists to identify the circuitry and cellular signals such as perceptions, emotions, motor functions, etc. [140]. There are two commonly used strategies of DREADDs: delivery-expression from genetically engineered mouse models and viral injection. The DREADDs-expressing viral vectors permit expression in an anatomically specific location [141]. NMDA receptors in long propriospinal neurons (LPSNs) that provide connections to the CST area [142] were modulated via genetic and chemogenetic tools in a mouse model with Th8 dorsal hemisection. Chemogenetic modulation led to the regrowth of CST axons competitively selecting their postsynaptic partners. It has been shown that the remodeling itself did not affect mature or uninjured circuits and enabled a functional recovery in mice [143].

Optogenetics is another approach in how to remodulate circuits after spinal cord injury. Optogenetics is a technique where neural activity is controlled with light by the genetic modification of light-sensitive proteins [144]. An AAV mediated expression of LMO3 (consists of Gaussia luciferase fused to Volvox channelrhodopsin 1) led to the locomotor recovery after SCI in the rat´s Th9 contusive model. The locomotor functional recovery was achieved due to the optogenetically induced neuronal plasticity of interneurons and motoneurons below the injury site, potential maintenance of neural networks, and improved inflammatory state [145].

### 4.3. Suppression of the Inhibitory Factors and Apoptosis

**Figure 5:**
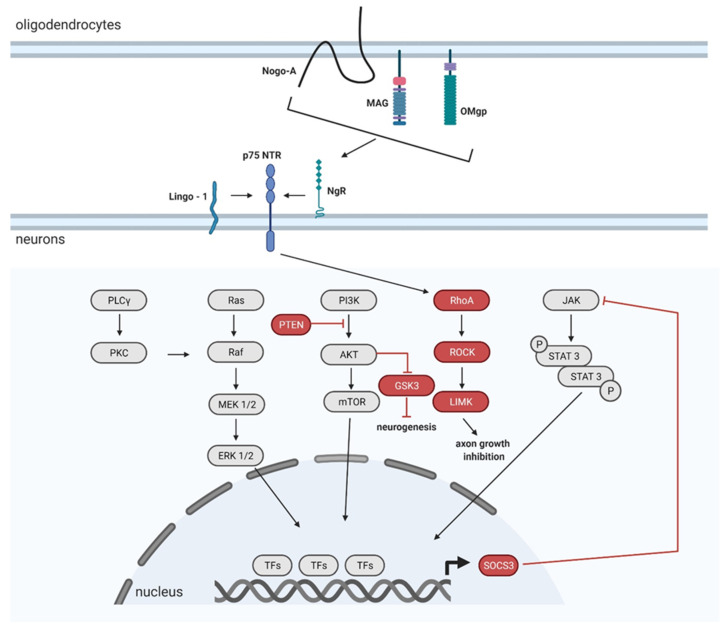
Schematic representation of experimentally inactivated targets mentioned in the text above. Inhibitors and TFs due to which CNS effort to regenerate fails are marked in red (Created with BioRender.com).

The transcriptional suppression of inhibitory molecules can provide the regeneration of adult neurons across the lesion site, and inactivating genes in vivo became an important technique for establishing their function in the adult CNS (Figure 5). The best way of how to effectively knock down certain genes is by small hairpin RNA (shRNA). This artificial RNA molecule can silence target gene expression via RNA interference [146]. Another approach in how to inactivate genes involves the use of Cre recombinase to remove the loxP-flanked segments of DNA [147].

Injured axons possess an extremely limited ability to regenerate within the CNS. The regeneration is mainly limited by 3 inhibitory proteins, oligodendrocyte-myelin glycoprotein (OMgp), myelin-associated glycoprotein (MAG), and neurite outgrowth inhibitor (Nogo), which are present in the injured microenvironment. All of them bind to the axonal Nogo receptor (NgR) [148]. The interaction between NgR and OMgp/MAG/Nogo activates the Rho/Rho-associated protein kinase (ROCK) signaling pathway in order to impact actin cytoskeletal dynamics, which leads to the collapse and retraction of the growing cones [149]. It has also been shown that an AAV mediated downregulation via AAV-shRNA of ROCK2 and LIM domain kinase 1 (LIMK1) led to an increased neurite growth after optical nerve axotomy, although the downregulation of just ROCK2 decreased neuronal death in the lesion site and attenuated axonal degeneration [150]. Furthermore, another study demonstrated that the specific knockdown of RhoA mediated by AAV increased neuronal survival after optic nerve axotomy besides neurite outgrowth and axonal regeneration after optic nerve crush [151].

Glycogen synthase kinase-3β (GSK-3β) is also an important axon growth-inhibitory factor involved in the Nogo-66 signaling pathway [152]. GSK-3β has been suggested as a key molecule downstream of the PI3K pathway to control axon growth and/or branching [153]. The stereotactic injection into the sensorimotor cortex of shRNA GSK-3β-AAV in Th8 spinal cord transected rats downregulated GSK-3β, and therefore promoted axonal regeneration by myelin inhibitor (Nogo-66) neutralization [154].

Phosphatase and tensin homolog (PTEN) blocks the growth and extension of adult neurons, as is known for the negative regulation of the mTOR pathway. mTOR downregulation in adulthood and after axonal injury inhibits the regenerative potential of damaged neurons [155]. PTEN converts phosphatidylinositol-3,4,5-triphosphate (PIP3) to phosphatidylinositol-4,5-bisphosphate (PIP2) [156]. The Pi3K/AKT/mTOR pathway is activated when PI3K converts PIP2 to PIP3; PIP3 further leads to AKT, and indirectly, to mTOR activation [157]. PTEN gene silencing prevents PIP3 to PIP2 conversion, thereby disrupting mTOR inhibition. In addition, it leads to an increased regrowth of the CST, after dorsal crush injury of the Th8 level in the mouse model [158]. PTEN deletion may also be mediated via the AVV-Cre system. The Cre-lox system is a specific system for controlling gene expression. Basically, Cre recombinase recognizes (locus of X-over P1) loxP DNA sites. It depends on the orientation and location of the loxP to determine if the genes will be activated/inactivated when Cre is present. The deletion can be achieved when loxP sites are in the same direction. The certain sequence between the loxP sites is further deleted and is not maintained afterwards. In the mouse (PTEN*^loxP/loxP^* strain C;129S4-Pten*^tm1Hwu/J^*) model with C5 contusion injury, AAV-PTEN-Cre enhanced recovery of the forepaw grasping and gripping function, and increased regenerative growth of injured CST axons [159]. Similarly, co-deletion of PTEN and the cortical suppressor of cytokine signaling 3 (SOCS3) resulted in robust CST sprouting with an appearing functional recovery of forelimb movements in mice after pyramidotomy [160]. In addition, the simultaneous deletion of both PTEN and SOCS3 after optic nerve injury in mice enables robust and sustained axon regeneration by regulating two independent synergic pathways (JAK/STAT and mTOR) promoting enhanced axon regeneration [161]. In addition, AAV mediated Cre deletion of SOCS3 promotes the sprouting of uninjured CST axons to the denervated spinal cord, in the mouse unilateral pyramidotomy model [162]. Neuronal expression of SOCS3 inhibits STAT3 and it contributes to excitotoxic neuronal death in vitro [163]. Homozygous conditional SOCS3 mutants (SOCS3^f/f^) injected with Cre-expressing adeno-associated virus into the sensorimotor cortex after unilateral pyramidotomy, showed increased sprouting six weeks after the injury. The mechanism may underlie the SOCS3 regulated compensation of the sprouting of spared CST axons [163].

The secondary response to the SCI alters several cellular and metabolic conditions, namely, lipid peroxidation, free radical production, vascular changes, mitochondrial dysfunction, and apoptosis [162]. The AAV-mediated overexpression of neuroglobin after Th12 chloral hydrate induced injury in Wistar rats reduced the release of cytochrome c, decreased apoptosis in the lesion site, and renormalized levels of oxidative markers [164]. The neuroglobin is a globin hemoprotein, involved in the oxygen homeostasis of a cellular system. Neuroglobin binds to oxygen with greater affinity than haemoglobin and plays a protective role after CNS injury by providing oxygen under hypoxic and ischemic conditions; it reduces oxidative stress and improves the functions of mitochondria [165].

### 4.4. The Modification of Glial Scar, Extracellular Matrix, and Cytoskeleton

In recent years, it has been shown that the glial scar plays a dual role, protective and inhibitory, in recovery after SCI. The scar is mainly formed by scar-forming astrocytes. There are several approaches for how to modify the glial scar formation [40,41,166]. One of them is the direct reprogramming of astrocytes into neurons, using a single transcription factor Sry-related HMG-box 2 (Sox2) delivered by AAV viral vector. Sox2 is a strong transcriptional activator that can reprogram resident astrocytes into functional neurons [167]. After an injection of Sox2-AAV, the astrocytes turned into neurons and it moderately attenuates the density of the glial scar without interrupting its integrity, and, in addition, it replenishes the neuronal loss at the same time. To postpone the preprogramming process, the viral vector injection was delayed and applied one week after SCI. The experiment was carried out in a mouse compression model of SCI. The glial scar formation was halted before the chronic phase began. It means that glial scar kept its protective role, but its inhibitory role was attenuated [168].

Additionally, astrocytes can also be converted into neurons via gene therapy, leading to neurogenic differentiation via 1 (NeuroD1) expression. NeuroD1 is a neural transcription factor which mediates the direct conversion of reactive glial cells into functional neurons. The remaining astrocytes proliferated to repopulate themselves. The astrocyte converted neurons were fully functional [169,170]. The NeuroD1 AAV-mediated expression led to the direct conversion of astrocytes into neurons in the injury sites which related to glial scar reduction followed by many effects, such as reduction of microglia and neuroinflammation, increase of neuronal dendrites and synaptic density, restoration of a blood–brain-barrier, and rebalancing neuron to astrocyte ratio. These findings were observed in a mouse model of stab injury [171].

Reactive astrocytes and other glial and non-neural cells in the glial scar secrete chondroitin sulfate proteoglycans (CSPGs) [41]. CSPGs play one of the major roles in the mechanical barrier of axonal outgrowth and regeneration [172]. CSPGs can be enzymatically degraded by bacterial enzyme chondroitinase ABC (ChABC) [173]. It has already been proven in SCI preclinical studies that ChABC improves regeneration and functional recovery [174,175]. However, ChABC has a short half-life and is not very thermally stable, which means it has to be administrated repeatedly [176]. Previous study has demonstrated that the viral delivery of ChABC in mammalian neurons can successfully degrade perineuronal nets (PNNs) for a longer period in a rat model of SCI [177,178]. PNNs are a layer of condensed extracellular matrix enwrapping neuronal somas and dendrites. PNNs appear late in development when the critical period closes, and CNS is no longer open for the plasticity. PNNs play a crucial role in the control of CNS plasticity as well as regeneration after the CNS injury. Their removal is an option for how to reactivate plasticity in adult CNS [179].

Recent findings have shown that there was an effort to target cell-types with ChABC selectively in vivo using adeno associated viral vector. The spatial specificity was achieved under the control of the Cre-LoxP system. ChABC was synthetized in Cre-expressing cells. This method allowed the cell-specific targeting of ChABC and long-term degradation of PNNs and may become an appropriate strategy for the treatment of neurodevelopmental disorders associated with PNN pathology [180], such as schizophrenia [181].

Another option for how to catalyze the proteolysis of CSPGs protein cores is a human enzyme, a disintegrin and metalloproteinase with thrombospondin motifs 4 (ADAMTS4) [182]. An AAV-mediated ADAMTS4 expression in the spinal cord injured site has shown functional recovery. The ADAMTS4 treated rats had a decreased lesion site and an increased axonal sprouting of corticospinal tract, after contusive injury [183].

Semaphorin 3A (Sema3A) is another ECM molecule that contributes to the inhibition of axonal regeneration by affecting the microtubules and the actin cytoskeleton [174]. Sema3A interacts with the membrane-bound receptor, Neropilin-1 (Nrp1), and its coreceptor, Plexin A. This interaction activates Rac1, a Rho family small G protein, and leads to axonal growth inhibition as previously mentioned in the text. The results have shown that the suppression of Nrp1 in mice via viral vector injection, immediately following SCI, led to an increased number of collaterals compared to that of the control group [184].

Glial scar-forming is the result of a multicellular response to an injury involving astrocytes, microglia, macrophages, oligodendrocyte progenitors, fibroblasts, leptomeningeal cells, and Schwann cells [185,186]. One of the major CSPG components after SCI is NG2, which is expressed by macrophages and oligodendrocytes progenitors [187]. This integral membrane CSPG, NG2, limits the growth of axons after injury. The expression of NG2 can be decreased with AAV-L1 therapy [188]. In a rodent model of SCI, L1 promotes neurite outgrowth, neuronal migration, and finally yet importantly, neuronal survival, in a self-binding manner [189,190,191,192]. Normally, L1 is abundantly expressed by growth cones and axons, but after SCI L1 expression, it is downregulated [193,194].

The administration of AAV-L1 in the mice Th7-T9 compressive SCI model improved motor functional recovery, enhanced axonal regeneration, reduced astroglial proliferation by reducing levels of Glial fibrillary acidic protein (GFAP) and NG2, and affected the signal transduction mechanism via an elevated level of phosphorylated CREB and MAPK [188]. The application of a soluble chimeric dimer was made by linking mouse L1 to human Fc neurite outgrowth and neuronal adhesion. L1 is one of the cell adhesion molecules and it is evolutionarily well conserved from Fugu fish to human [195]. Fc-fusion protein is composed of an immunoglobin Fc domain directly binding to another peptide. The human Fc plays an important role in the chimeric dimer from the biophysical perspective. The Fc domain can improve the stability and solubility of the partner molecule, both in vivo and in vitro [196]. This dimer also contributes to the protective functions of the immune system [197], and led to locomotor recovery in adult rats after contusion spinal cord injury [193].

The dynamics and organization of the cytoskeleton also plays a key role in axonal regeneration. Injured axons may become growth-incompetent due to changes in the axonal cytoskeleton, but the cytoskeletal modulation can change the dynamics of the injured axons, and alter the growth-incompetent cones into growth-competent ones [198]. The axonal growth after SCI may be stimulated by profilin 1 (Pfn1). Pfn1 is a small actin-binding protein involved in the regulation of actin assembly during development [199]. The AAV delivery of constitutively active Pfn1 increased axonal regeneration through the inhibitory glial scar after SCI in rodent models as well as promoting the axonal regeneration and functional recovery of the injured sciatic nerve [199].

As previously stated, axonal regeneration after SCI is blocked by the inhibitory environment of the glial scar, and by the developmental loss of regenerative potential. In cell migration, there must be an adhesive molecule that can recognize a ligand in the environment, and which is linked to signaling and cytoskeletal mechanisms. In the development of the CNS, integrins play a crucial role as adhesive molecules. Ligands for the integrin alpha-9 (ITG9) are the Tenascin C (TN-C), the main ECM glycoprotein of the adult CNS, which is upregulated at the lesion site [200]. An AAV mediated overexpression of ITG9 in DRG neurons resulted in a significant ingrowth of axons into the lesion site after a dorsal column lesion [201]. However, it has been shown that the integrin mediated axonal regeneration is inactivated, due to the presence of inhibitory molecules such as Nogo and CSPGs [202,203]. Integrins are heterodimers containing one α- and one β-subunit [204]. The integrin activation can be enhanced by kindlin-1 binding to the β-subunit of the integrin heterodimer [205]. The administration of AAV-kindlin-1 leads to the overexpression of kindlin-1 in DRG neurons activating integrin signaling. This activation led to significantly more axons entering the spinal cord after dorsal root injury [201]. In addition, the synergistic effect of ITG9 and kindlin-1 has been shown in sensory axon regeneration after a C5-C8 root crush, when an AAV injection (ITG9+K1) into C6 and C7 led to anatomical and electrophysiological evidence of axonal reconnection in the animal model, as well as behavioral recovery [206].

## 5. Conclusions

In the past few years, gene therapy has been shown as a promising solution for SCI treatment in experimental models. An enhanced knowledge of the onset and progression of SCI will enable the choice of a target (a combination of well-chosen target genes in some cases) for the viral construct to: recreate the developmental environment of the CNS, remodulate neuronal circuits or reactivate some of the dormant circuits, suppress the inhibitory factors that emerged within the injury response as well as the cellular death pathways, and modulate glial scar, extracellular matrix, and the cytoskeleton—with the potential to be translated into clinical trials. The AAV-mediated gene therapy depends on the model used in the experiment and the route of the viral administration. Besides, the AAV vector efficiency also relies on cellular tropism and the vector promoter [207].

Although the therapy may seem to be successful in a rodent model, the transfer into clinical trials may not be currently feasible. One of the main obstacles preventing the successful transfer of a preclinical rodent AAV-driven gene therapy into patients is the fact that we cannot precisely predict the effect of a certain gene transfer in human patients. The AAV-mediated gene therapy for SCI must be optimized from the perspective of relevant model development; determination of the optimal route of administration, dose (e.g., the high systematic doses were shown to be toxic), titer, tissue target, and the frequency of application; optimization of the choice of the capsid and genome conformation; an understanding of how the immune system works. It has been shown that rodents are not natural hosts for AAVs [82]. Animals have a different immune response to the rAAVs. Human patients might have neutralizing antibodies against viral capsid. The possible solution could be the selection of patients with low neutralizing antibodies, using modified capsids (seroprevalent, not-cross-reactive, etc.) or administering immunosuppressive drugs. CD8+ T cell-mediated cytotoxic immune response can be reduced by decreasing the vector doses and/or removing the empty capsids from vector preparations [208]. The preexisting immunity in individuals may lead to a lower level of transgene expression. Once the immune response is better understood, we may be able to improve our knowledge and solve the gene therapy for SCI or other CNS disorders. Lastly, the considerable limitation of transferability may occur within the gene therapy timing, while planning to influence many of the processes involved in the secondary phase of SCI. Under experimental conditions, the AAVs can even be administered before the SCI to initiate the expression of the target genes, as rAAV needs at least three weeks to start its expression, which is not possible in human clinics. The time window for the treatment after SCI is a particularly important parameter and must be considered when designing the experimental concept of the studies; this is dependent on the target genes that will be influenced and the pathophysiological events that will be manipulated.

To conclude, the main advantages are biosafety rating, low immunogenicity, and the ability to interact with divided and non-divided cells, and finally yet importantly, the long-term viral expression. The drawback of the AAV-mediated gene transfer should be mentioned as well. Viral vectors have their own limited capacity. It means that large genes cannot be transferred. Neutralizing antibodies against AAV may be generated in the host and the cure effect of AAV-mediated gene therapy becomes attenuated. In addition, the time needed for AAV vector expression is rather long (three weeks) and can be an obstacle in acute or traumatic conditions, where a quick response is needed.

As afore-mentioned, several studies have demonstrated that there are thousands of viral variants naturally existing and genetically prepared. All these variants trigger a different immune response in different animal models. The real breakthrough in target gene vector, shown in rodent models, should be tested in larger animal models or/and NHPs to have a chance to start the clinical trial process in human SCI patients [209]. Future studies are necessary for the safety evaluation of this treatment approach. However, we believe, as progress continues in optimizing transgene design, delivery, and vectors, the prospects of gene therapy for spinal cord injury will look more promising.

## Figures and Tables

**Table 1:** Summary of clinical trials listed on website https://clinicaltrials.gov/ cited on 9 February 2021 focused on AAV mediated gene therapy in neurodegenerative disorders.

Neurodegenerative Disorder	Viral Vector	Gene Transfer into	Phase of Clinical Trials
Alzheimer’s Disease	AAV-hTERT	IT, IV administration	I
	AAV-NGF	Basal forebrain	I
	AAVrh.10hAPOE2	CNS/CSF	I
	AAV2-GDNF	putamen	I
Batten Disease	AAV2CUhCLN2	CNS	I
	AAV9-CLN3	lumbar IT	II
	AAVrh.10CUCLN2	directly to the CNS	II
Charcot–Marie–Tooth Neuropathy Type 1A	scAAV1.tMCK.NTF3	IM	I, II
Frontotemporal Dementia	AAV9-PGRN	cisterna magna	I, II
Huntington’s Disease	rAAV5-miHTT	striatum	I, II
Late Infantile Neuronal Ceroid Lipofuscinosis	AAV9-CLN3	lumbar IT	II
	AAVrh.10CUCLN2	CNS	II
	AAV2CUhCLN2	brain	I
	scAAV9.CB.CLN6	IT	I, II
Leber Hereditary Optic Neuropathy	rAAV2/2-ND4	intravitreal administration	III
	scAAV2-P1ND4v2		I
Multiple System Atrophy	AAV2-GDNF	putamen	I
Parkinson’s Disease	AAV-GAD	nc. subthalamicus	I, II
	AAV2-GDNF	damaged brain area	I
	AAV-hAADC-2	striatum, putamen	I
	AAV2-AADC	striatum	I
	AAV2-NTN	putamen, subst. nigra	I, II
Spinal Muscular Atrophy	AAV9-CAG-SMN	IV infusion	III
	AAV9-CAG-SMN	IT/IV administration	I
	AAV9-CAG-SMN with modified	IV infusion	III
	ITR		
	AVXS-101(AAV9-CAG-SMN)	IV	Approved for marketing (2019)
Wilson’s Disease	AAV-ATP7Bminigene	IV	I, II

Data found under keywords: AAV, Neuro-Degenerative Disease.
